# ‘No, you should not beat our child because he will become aggressive:’ Applying a multi-method approach to explore intergenerational transmission of parenting practices

**DOI:** 10.1371/journal.pone.0258306

**Published:** 2021-10-07

**Authors:** Varalakshmi Chandra Sekaran, Ajay Bailey, Veena Ganesh Kamath, Lena Ashok, Syam K. Ravindran, Asha Kamath, Asha Hegde

**Affiliations:** 1 Department of Community Medicine, Melaka Manipal Medical College (Manipal Campus), Manipal Academy of Higher Education, Manipal, India; 2 Transdisciplinary Centre for Qualitative Methods, Prasanna School of Public Health, Manipal Academy of Higher Education, Manipal, India; 3 Faculty of Geosciences, Department of Human Geography and Spatial Planning, International Development Studies, Utrecht University, Utrecht, Netherlands; 4 Department of Community Medicine, Kasturba Medical College (Manipal), Manipal Academy of Higher Education, Manipal, India; 5 Department of Global Health, Prasanna School of Public Health, Manipal Academy of Higher Education, Manipal, India; 6 Department of Clinical Psychology, Manipal College of Health Professions, Manipal Academy of Higher Education, Manipal, India; 7 Department of Data Science, Prasanna School of Public Health, Manipal Academy of Higher Education, Manipal, India; 8 Department of Paediatrics, Melaka Manipal Medical College (Manipal Campus), Manipal Academy of Higher Education, Manipal, India; St John’s University, UNITED KINGDOM

## Abstract

**Background:**

Exploring the cultural context of intergenerational continuity of warm and harsh parenting informs parents motivations to adopt specific parenting behaviours.

**Objective:**

Parents’ perceptions of being parented in the past and their current parenting as well as adolescents’ perceptions of current parenting were explored applying a multi-method approach.

**Methods:**

Following written informed consent, a total of 24 interviews with 10 families (dyads of 14 parents and ten adolescents) from Udupi taluk in southern India was conducted. In the first stage, in-depth interviews were conducted with parent participants (Generation 1 (G1)) and in the second stage, adolescents (Generation 2 (G2)) participated in the photovoice component. Multiple forms of data including photographs, journals and interviews facilitated using the SHOWeD model were collected and were analysed thematically using ATLAS.ti(v.8).

**Results:**

Subtle changes in reinforcing culture-specific gender norms between generations were elicited. Differences in communication, granting autonomy to female adolescents, and in disciplining methods between G1 and G2 were observed. Warm parenting was transmitted between generations while harsh parenting in G1 in the presence of external social support was discarded in favor of warm parenting in G2.

**Conclusion:**

We provide evidence for perceptions of parenting and adolescent behaviors across two generations. Transmission of warm parenting and interruption in the cycle of harsh parenting in the presence of external social support were significant findings. Related theoretical and methodological applications are discussed.

## Introduction

An optimal family environment allows for the individual to grow and develop into functioning autonomous individuals [1–3]. Conversely, a poor family environment and parenting practices in particular, may lay the foundation for long-term adverse outcomes among maturing adolescents [4–6]. The 2030 agenda of the Sustainable Development Goals (SDGs) includes 35 indicators having a direct bearing on children’s well-being, emphasizing the importance of optimal outcomes among them [7]. In exploring parenting practices, there is a time-honoured hypothesis that the way one generation experienced parenting themselves are transmitted to the next generation. Intergenerational transmission of parenting [8–10] may occur through modelling of parenting behaviour as described under the social learning theory [11] and may also be influenced by the attachment theory [12]. The social learning theory proposed by Bandura A., [13] emphasized the importance of modelling emotional reactions, attitudes and behaviors by observing others within the social context. He proposed that learning may occur on observing the behavior of others and the outcomes of those behaviors. Cognition also had a role in learning, he stated, and that learning can occur without a change in behavior. The theory also states that ‘reinforcement’ and ‘punishment’ may have a bearing on whether the individual exhibits the learned behavior. In keeping with this theory, intergenerational transmission of parenting may occur through children modelling and imitating their parents’ behaviors. The attachment theory as proposed by Bowlby J., [12,14] states that secure attachments with caregivers lay a foundation that enables children to form secure relationships themselves. Experiencing secure relationships with parents may provide an internal working model for children providing a base on which to develop their own behaviors and exhibit social competency [15]. On the other hands, parents who are dismissive of negative experiences in childhood or have a preoccupation with their own attachment experiences in the past may provide a less secure attachment experience to their children. However, past experiences may be influenced by later secure relationships and the social context. Traumatic experiences in the past have been found to interfere with attachment between parents and their children [16].

Parenting literature estimates that 35%-45% of parenting behavior transmits between generations [17]. Traditionally, parenting has been described by Baumrind, D. [18] as consisting of three styles which were further described by Maccoby and Martin [19] as corresponding to higher or lower degrees of ‘warmth’ or ‘responsiveness’ and ‘demandingness’ or ‘control.’ Baumrind has characterised warmth as “affective warmth, cognitive responsiveness, attachment and bonding, unconditional acceptance, sensitive attunement, involvement, and reciprocity [20].” Warmth expressed by the parent toward their child may be exhibited as praise, support, encouragement and affection [21–23]. Warm parenting constituting parental involvement and exhibiting nurturing behavior towards their wards has consistently been found to be effective in the optimal development of children and adolescents [24,25]. These experiences of family cohesion, open communication, and lower conflict promoted similar behaviors when the children themselves became parents [8,9,24,26]. On the other hand, hostile and punitive parenting was also found to be transmitted between generations [27,28]. Harsh parenting is a negative form of control through assertion of power by the parent toward the child. This may be expressed through hostility including corporal punishment towards the child or adolescent and may be expressed through anger, hitting, kicking, pinching, cursing, criticizing or using sarcasm [29–32]. This form of parenting has been found to adversely affect child and adolescent wellbeing and may lead to the development of internalizing or externalizing behaviors [30,33]. However, harsh parenting behaviors from one generation are not inevitably transmitted to the next as seen from literature [27,34,35] and the cycle of maltreatment may be broken in the presence of external supportive relationships.

Literature related to family studies [36–41] in India is growing but several gaps in understanding specific dimensions of parenting, transmission of parenting from one generation to the next, or the cultural impact on parenting do exist. Traditionally parenting in India reflects the collectivistic hierarchical nature of society and emphasizes interdependency, deference to elders, obedience and conformity [42]. Strong family bonds are encouraged ensuring that the younger generation shoulders the responsibility of caring for vulnerable dependent family members. In recent decades, there has been a gradual move towards becoming an industrialized society. This has resulted in structural and functional changes percolating to the basic family unit. The traditional joint family consisting of at least two generations of related kin living together, has given way to various types of families including nuclear and extended families [36]. Consequent to these changes, reciprocal alterations in the behavioral and social interactions within the family are to be expected [43]. While there is evidence on parenting in the western context [27,34], literature on warm/harsh parenting and its transmission between generations is scarce in the global South and in the Indian context. The changes within the family are becoming evident in recent decades with urbanization. India traditionally is a collectivistic culture but changes in family structure, reduction in family size, etc., could influence family relationships including parent-child interactions [35]. With changing socio-cultural aspects, indigenous research would likely add nuanced findings on our understanding of integrational continuity of parenting, the reasons for why parents choose to parent the way they do and how adolescents perceive them.

In our study, we aimed at understanding parenting experiences between two generations in the Indian context. We examined childhood experiences of being parented in Generation 1 (G1) and parenting the present generation (G2) from the perspective of both the adolescents as well as their parents. Secondly, we explored whether warm and harsh parenting experienced in G1 is inevitably repeated in the next generation (G2) and the reasons that may contribute to changes in parenting behavior between generations were also explored.

## Materials and methods

This community-based participatory research study employed a multi-method approach involving qualitative and photovoice methodology to explore transmission of parenting across two generations. Consent was informed and written consent was obtained prior to data collection.

### Participants

The study was conducted at Udupi and Brahmavara taluks of Udupi, a coastal district in southern India. Following clearance from the Manipal University Ethics Committee (MUEC/013/2017), participants were identified with the help of community health workers from lists maintained at anganwadi centres (community health centres) under a government initiative. Parents and their adolescent child aged between 14–19 years of age who had resided within the same household for least three months prior to the interviews were invited to participate. We used purposive sampling method, which is a form of nonprobability sampling. In order to obtain a representative sample of the population being studied, purposive sampling was employed [44]. Using this method, thirteen families were approached of whom 10 two-parent families consented to participate. An attempt was made to include both parents and one adolescent from the same family in order to understand the intergenerational transmission of parenting. When more than one adolescent was present within the same family fulfilling the inclusion criteria, the oldest was invited to participate. Both parents and one adolescent from five families participated and among the five remaining families, four mothers and one father agreed to participate. Fathers were aged between 46–55 years and mothers were aged between 38–52 years of age. Five of the mothers were employed and the rest were homemakers. All of the fathers except one were employed. Among the adolescent participants, five were male and five were female. Of them, three were single children while the rest had at least one sibling. The family-type included five nuclear and five joint families with at least one grandparent living with them. All of the families belong to the middle class based on the BG Prasad scale, 2017. This scale measures social class based on the monthly per-capita family income [45–47]. Six of the families resided in urban areas while four were from rural areas. The study spanned between July 2017 to June 2018.

### Data collection: In-depth interviews for parents

The data collection proceeded sequentially with the parents being interviewed following which the adolescents were recruited into the photovoice component of the study. In the first stage of data collection, parents (G1) were approached at their convenience in their homes or at the local anganwadi centre. An IDI guide with about 18 questions and probes was developed based on literature for the parent participants. The interview guide was structured to have opening questions, key questions and fade out questions [48]. The key questions probed regarding the experiences of parents during their adolescence including family structure, parental involvement, disciplining methods, autonomy and decision making, and similarities or differences in currently parenting their adolescent children in comparison with their own experiences. Some of the questions included: What was the general atmosphere of your family? Were you able to communicate with your father/mother on things that you thought were important to you? If you disagreed with your parents on some things, were you able to express it? How would your mother react/How would your father react? How would your parents react when you did something right? Do you feel things are the same or different in the way that you parent your adolescent child/children compared with how you were parented? Content validity was performed with the help of professionals in the field of Cultural Anthropology, Community Medicine, Social Work, and Public Health. The individuals in this manuscript have provided written informed consent (as outlined in PLOS consent form) to publish these case details. Individual interviews, lasting about 45–60 minutes, were conducted at a convenient time. The primary researcher conducted the interviews which were recorded and notes were maintained. The guide was framed with opening, key and closing questions. Based on the preference of the participants, the interviews were conducted in either English or Kannada (local language). The audio recordings were transcribed verbatim. Translation into English was performed by a native Kannada speaker knowledgeable in English.

#### Photo-narratives for adolescents

In the second stage, adolescents (G2) were invited to participate in the photovoice component of the study. During the initial contact with the adolescents, the researchers described the aim of the study, i.e., to understand how adolescents viewed their world with regard to family and other relationships. Over a period of a week, they were required to take photographs of their routine interactions with family members and peers, their hobbies/interests, activities, as well as photographs related to their likes and dislikes. Based on these photographs, interactions with family members were probed into. The participant-led methodology was explained to the adolescents where narratives are built around visual data in the form of photographs and journal data [49–51] pertaining to their interactions with family members. The photographs served as a creative and fun vehicle which encourages adolescent participants to convey their life experiences. They were also encouraged to maintain a journal of their interactions with family members over the period of a week. Parental consent, adolescent assent and photography release forms were obtained prior to data collection. With the photographs being used as prompts for the interviews, the SHOWeD model as proposed by Wang et al., [48] was followed with audio recording. The SHOWed methodology provides probes to explore the meanings of the submitted photographs using these prompts: Using the SHOWed prompt for the photographs: What do you *see* here? What is really *happening* here? How does this relate to *your* lives? *Why* does this concern, situation, or strength exist? How can we become *empowered* through our new understanding? And, what can we *do*?

Interviews were conducted to probe into these questions with the photographs as prompts. The key questions corresponded to the questions on the IDI guide for the parents. The broad areas explored corresponded to those explored among the parent participants and included questions related to family relationships, parental involvement in the adolescent’s social life, disciplining methods, relationship with other family members/peers and adolescent mental health needs. Transcription of the interviews in English were carried out as close as possible to the interview dates. Overall, the study data included IDIs from 14 parents and 10 adolescents along with data from 10 journals and 62 photographs. Access to the study data was limited to the research team.

### Data analysis process

ATLAS.ti (v8) was used to analyse the data. For each of the participants, the data was compiled into a single file prior to analysis. The themes were developed based on the data which included interviews with the parents and from the multiple forms of data collected from adolescents including photographs, journal data and interviews using the SHOWed methodology. Multiple data capturing methods lent to the validity of the data. Two cycles of coding were performed. Primary codes were identified both inductively and deductively while in the second cycle of coding, sub-themes and themes that emerged from the data were developed. Thematic analysis was performed [48]. The themes and sub-themes are listed in able 1 and structure our discussion of the results.

## Results

The broader themes were two-fold with several sub-themes. The broad themes include (i) Parents’ experiences of experiencing parenting during childhood and adolescence and (ii) Parenting the current generation of adolescents as presented in Table 1 and the participant profile is presented in Table 2.

**Table 1:** Themes and subthemes.

Themes	Subthemes
Parents’ experiences of being parented during childhood and adolescence	• Warm but strict parenting in the past generation • Parents’ experiences of harsh parenting • Drivers to adopt or reject punitive forms of parenting for their own adolescent children
Parenting the current generation of adolescents	• Warm parenting transmitting across generations • Breaking the cycle of harsh parenting

**Table 2:** Participant profile.

Family no.	Parent participant	Age (years)	G1*	Adolescent participant	Age (years)	Urban (U)/Rural (R)	Family type (Current)^#^	G2**
1	F1	47	H	B1	15	U	Joint (F/M/)^**#**^	W
	M1	44	W					
2	F2	46	W	G2	15	R	Joint (F/M/)^**#**^	W
	M2	38	H					
3	F3	53	H	B3	17	U	Joint (F/M/S)	W
	M3	50	W					
4	F4	51	H	B4	14	U	Nuclear (F/M)^**#**^	W
	M4	46	H					
5	F5	53	H	G5	16	U	Joint (F/M/Br)	W
	M5	50	W					
6	F6	55	H	G6	15	U	Nuclear (F/M/Br)	W
7	M6	52	H	B6	19	U	Nuclear (F/M/Br)	W
8	M7	45	W	G7	14	R	Nuclear (F/M/Br)	W
9	M8	39	W	G8	16	R	Joint (F/M/S)	W
10	M9	52	H	B9	18	U	Nuclear (F/M/Br)	W

H–Harsh parenting/W-Warm parenting.

#—Single children/F-Father/M-Mother/B-Boy/G-Girl/Bi-Brother/S-Sister.

### Parents’ experiences of being parented during childhood and adolescence

Table 3 provides a concise view on the parenting and adolescent experiences at G1 and G2 compares family structure, parenting practices, adolescent perceptions and activities as well as differences observed between G1 and G2.

**Table 3:** Major findings on parenting and adolescent behaviors between generations.

Parenting towards G1	Parenting towards G2
Structure: Largely joint families	Structure: Largely nuclear families
Parents focused on being providers and was not approached consciously	Parents are keen on consciously parenting their children
Mothers were generally the go-to person for advice	Bonds between adolescents and parents paved the way for both parents to become confidants
Fathers enforced discipline within the household including corporal methods	Both parents shared decisions related to parenting adolescents including disciplining methods and non-corporal methods of disciplining such as using verbal commands and taking away privileges were practiced.
Lower acceptance and limited communication between parent-adolescent dyad	Communication between the adolescent and parents was higher in comparison
Academic achievement was emphasized on	Academic achievement was emphasized on
**Adolescent experiences in G1**	**Adolescent experiences in G2**
Male adolescents experienced higher autonomy	Male and female adolescents in urban areas both enjoyed autonomy, however, concerns for female adolescents in terms of safety were voiced
Restrictions were higher towards female adolescents	Female adolescents experienced higher autonomy comparatively
Activities for male adolescents included indoor and outdoor activities	Activities for male and female adolescents included both indoor and outdoor activities among urban participants
Activities for female adolescents were largely indoors	Female adolescents were encouraged to get involved in activities that they enjoyed to a higher degree than in G1

The subthemes related to how parents experienced parenting during their own childhood and adolescence are presented below:

#### Warm but strict parenting in the first generation (G1)

During the IDIs, G1 parents noted that their parents primarily sought to fulfil their roles as providers. ‘Parenting’ was not dwelt upon. Most parent participants perceived that their parents had loved and cared for them, however, there was less engagement between them. A gamut of factors may exert their influence on how a child in a particular family within the social context is raised [52], and these determinants may be unique and distinctive for each family [53].

*“My mom was a warm person… we had agriculture…in the evenings*, *we used to eat together*, *say prayers*.*”* (M7, female parent, 45 years)

*But*.. *there was no importance for us at all…father used to love me so much but he had no time to give me*.*”* (M3, female parent, 50 years)

“*Since we were five children*, *there was not much acceptance you know*. *Even when we did something well*, *he (father) will say–good–that’s all*.*”* (F4, male parent, 51 years)

Across most interviews, it was observed that communication, active engagement, or acceptance of the adolescent was not emphasized on. There was a general perception that fathers were ‘strict’ and most parents during their adolescence had preferred to communicate with their mothers.

*“I used to speak with my mother*. *Father was very strict…otherwise*, *he is a nice person actually but at that time*, *I felt that he was very strict*.” (M2, female parent, 38 years)

Gender norms and distinctions between male and female children were reinforced at the level of the household in keeping with conventional societal and cultural expectations. For instance, the provision of higher autonomy for the male adolescents in comparison with the female adolescents clearly came through during the interviews.

*“My father’s influence was very good*. *Even though I lost my father when I was in my 10*^*th*^ *grade until then*, *he used to guide me in the right way*. *That was for me a big influence*. *He was okay with anything I did*. *I would just tell him what I did*. *He was never strict with me*. *He was an extremely friendly person*.*”* (F2, male parent, 46 years)

*“He (father) used to talk to me*. *There was no problem*. *I had more liberty compared to my brothers and sisters …as I was the eldest*. *He used to appreciate me more*. (F5, male parent, 55 years)

Parents enforced gender-appropriate behaviours including restricting outdoor activities or purchasing clothing for female adolescents while reinforcing traditional forms of dressing such as long skirts and blouse or frocks which were acceptable in comparison with clothes which resembled western clothing such as jeans pants or short skirts as presented in Table 3. Fathers were primarily responsible for enforcing discipline within the household. One female parent who had fond memories of her family did express frustration at not being able to exercise her autonomy.

*“He (father) will shout*. *If you wear some short clothes and all*, *he will really shout*, *‘What you are wearing*? *Wear proper clothes*.*’ Sometimes*, *I used to tell them about what I like but some things*, *I was not able to talk to them*.*”* (M7, female parent, 45 years)

*“I liked wearing jeans you know which he (father) doesn’t like*. *He does not tell me now…but I very*, *very clearly remember*, *he did not like me wearing jeans*. *So*, *I had not worn it for a long time*. *Once*, *I got married*, *then there were no issues*.*”* (M1, female parent, 44 years).

Among most female parents, the inability to express themselves appeared frequently and at times, it would lead to gaps in communication and conflict. As was evident, these restrictions extended beyond the adolescent years too for some participants. The larger societal discourse of maintaining the family honour rested and continues to be seen largely as contingent upon the girl’s or woman’s behaviour [54]. These traditional norms are deep-rooted and were reflected in the interviews.

#### Parents’ experiences of harsh parenting

Among our participants, four parent participants including three mothers and one of the fathers (G1), stated that they had experienced harsh parenting in their childhood and adolescence. They had frequently experienced shouting, yelling or the use of corporal punishment such as being slapped or being hit with a stick or a belt as presented in Table 3. All of them stated that their fathers had used these methods to punish them while their mothers had tried to protect them.

The case study presented in Table 4 is a composite of several parents’ experiences of harsh parenting. G1 participants generally lived in extended households with several relatives living within the same household. While some of them had been punished following a misdeed, in several other instances, there had been no identifiable triggers apart from alcohol consumption by the fathers. It was observed that the spousal disturbances would frequently spill over to the parent-child interactions. The memories of feeling helpless and shame associated with these episodes, overwhelmed some of our participants emotionally during the interviews with one participant stating she wished that her father was ‘dead.’ One of the fathers similarly stated that he associated his father with feelings of ‘fear’ which he carried through into his adulthood. Emotions such as feeling ‘aggressive’ and ‘angry’ at their punitive parent while at the same time experiencing feelings of ‘powerlessness’ were also frequently mentioned. They depended largely on their mothers for emotional support.

**Table 4:** Case study: 1 Rani’s experiences of harsh parenting.

Rani (not her real name) is from a town in Karnataka, India. She was the second among six siblings. She lived in an extended family with her parents, siblings and her father’s sisters. Their father worked as a contractor while her mother was a homemaker. She recounts her younger years with dread that several rules had to be followed else there would be severe consequences from her father. They were not encouraged to have friends and no one was allowed to visit their home. Her father frequently became violent after consuming alcohol. Conflicts were frequent and the household had been subjected to their father’s violence. She also held a fear of sexual abuse from her father and it troubled her greatly. The atmosphere within the home was very negative and all too frequently, the neighbors would have to step in and protect them from their father’s rage. Rani confided in friends and a few trusted teachers and they encouraged her to pursue higher education to help her become independent. She felt anger, shame and helplessness and the need to leave home and become independent grew very strong. She recounted that during the end of her father’s life, he had apologized to her for his harshness towards them.

*“I was so angry and wished he was dead*. *These were all issues that really troubled me*.*” (M9* female parent, 52 years)

These experiences of harshness had forced some of them to rely on external sources for support including trusted relatives, teachers, friends and neighbors which helped them cope and become independent as presented in Table 3. In the absence of adequate support, literature cites the emergence of mental health problems, poor scholastic achievement or engaging in delinquency as outcomes [55].

#### Drivers to adopt or reject punitive forms of parenting for their own adolescent children

To understand intergenerational transmission of parenting, probes were used to explore whether parents adopted the same forms of parenting as they had experienced. We observed consistently that parents who had experienced warm parenting, generally sought to do the same for their children.

“*I encourage her (daughter) in everything*. *First of all*, *I wanted her to be good*. *I am not worried much about her academics*. *I don’t expect too much discipline and all*. *She is very confident*. *My father was very friendly*. *I want to bring up my daughter the same way”* (F2, male parent, 53 years)

One interesting finding was with a female parent who had herself experienced warm parenting herself but stated that she was not averse to using corporal methods on her son. However, her husband who had experienced harsh parenting himself, did not agree that it was an acceptable form of punishment:

*I never physically*, *what you call beat my son*, *may be once or twice…*. *simply because my husband and mother-in-law did not believe in hurting him*. *My husband told me*: *‘No*, *you should not beat our child because he will become aggressive*.*’ He says when his father had beaten him and he had felt very aggressive and wanted to beat him back while I believe that once in a while it’s okay*. (M1, female parent, 44 years)

Contrasting this with the beliefs held by her husband, it was evident that he set the rules as a disciplinarian in the family.

*“I was punished as a child*, *so that was the reason why when it came to our parenting*, *there are some ground rules*. *Even when my wife violates them*, *sometimes I feel angry*. *I am completely against any sort of physical punishment*. *So*, *I would probably say that was the only ground rule which I think*, *I used for parenting*.*”* (F1, Male parent, 47 years)

Hence, based on her husband’s experiences, the mother acquiesced and deferred use of punitive measures on their son. This is in keeping with the societal norms of conceding to the male member of the household as having the decision-making authority in aspects of family matters and child-rearing. Exploring these facets of disciplining from the point of view of both parents, provided insight into reasons why parents chose to discipline their adolescents in the way that they did.

Parents looked to their own past experiences and the emotions that they had experienced which led them to either adopt or reject harsh disciplinary practices on their children. One of the female parents who had experienced harsh parenting emphasized that in parenting her adolescent son she had adopted an involved role as opposed to her own experiences. In describing her current parenting, she stated:

*“We have to be very close to our children*. *We have to understand them because this is the age when they need somebody close and in his case*, *he is an only child…*. *we try to give him the support in whatever way we can*.*”* (M4, female parent, 46 years)

It was evident from our findings that the experiences of harsh parenting led to a desire among these parents to disallow similar experiences for their own child.

### Parenting the current generation of adolescents (G2)

Adolescent participants were interviewed and their perceptions regarding current parenting approaches using the photovoice methodology provided data in the form of photographs, journal content and interviews for this component of the study.

#### Warm parenting transmitted across generations

**Fig 1:**
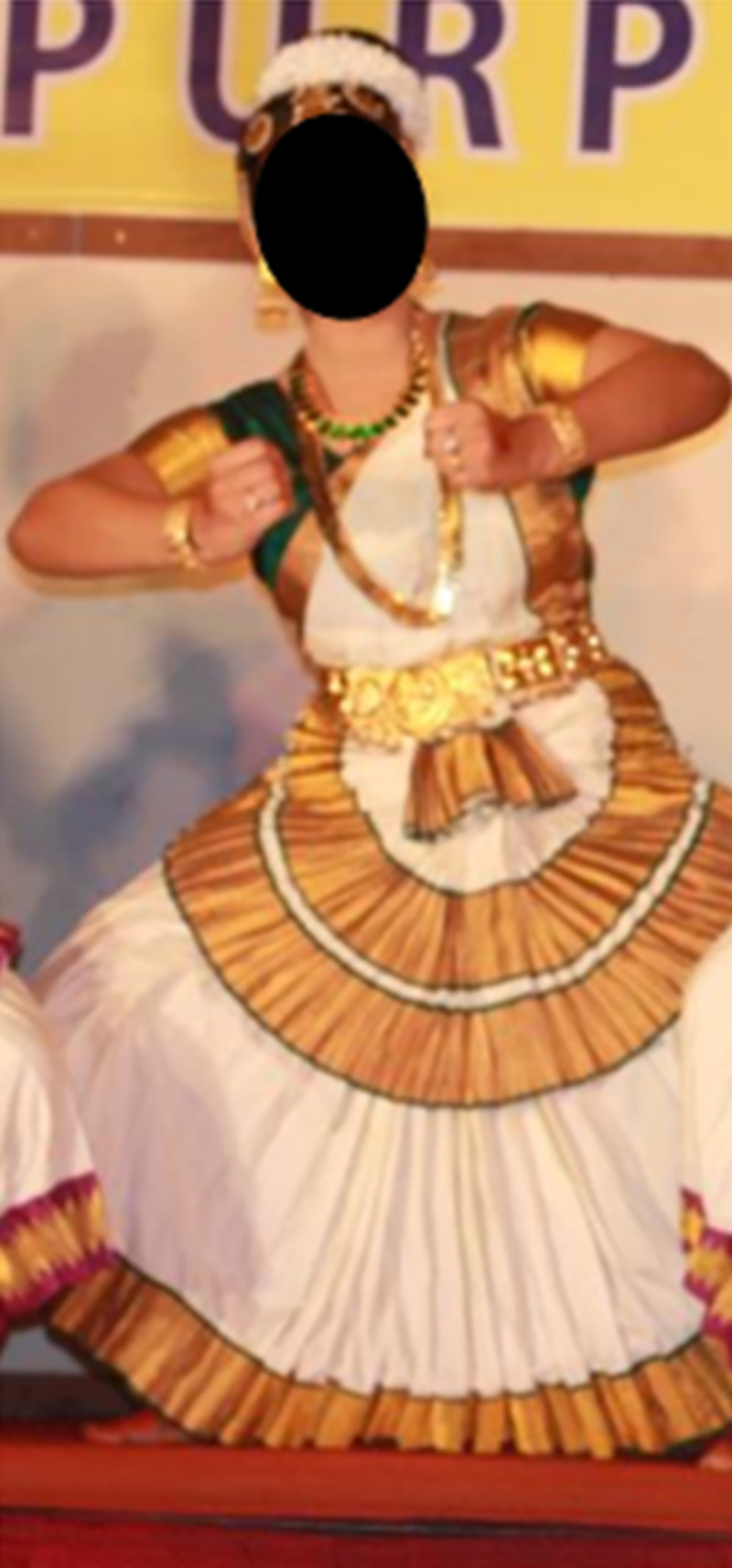
Dance is my passion—Mohiniyatam © G2, female, 15 years.

**Fig 2:**
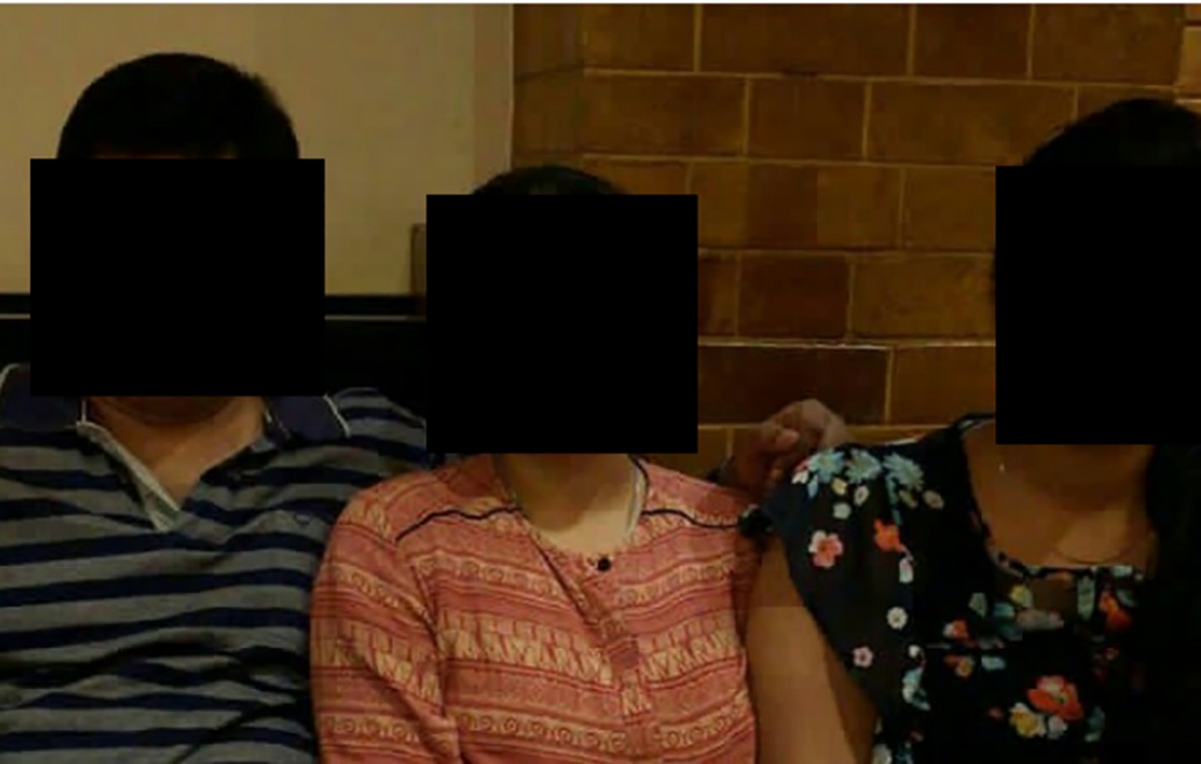
Friendly parents © G2, female, 15 years.

On comparing data from G1 and G2, we observed that adolescent children of parents who had experienced warm parenting, also experienced affectionate and caring parenting. Interestingly, in G2, parents provided opportunities and autonomy in relation to various activities to both male and female adolescents. Granting autonomy to male adolescents appeared to remain consistent across G1 and G2. However, differences towards female adolescents were observed. Our findings reflected a subtle shift in the manner in which young women wished to express their autonomy and parents were willing to grant higher autonomy in comparison with G1. We cite the experiences of a female adolescent who held a passion for dancing, stating:

*“Dance*..*is my passion*, *I love it*. *I started this dance form ‘Mohiniyatam’ when I was in senior kindergarten*. *It just makes me feel happy and encouraged*.” (G2, female adolescent, 15 years) Figs 1 and 2

Traditionally, female children and adolescents have had less privileges as compared to male children as observed in G1. In the case of this participant, her mother did share concerns, reflecting the existing social tension between empowering women and safety concerns which continues to remain in focus. Hence, the degree to which an adolescent girl could exercise her autonomy was laced with concerns thus:

*“Sometimes*, *I will have to disagree with him (husband) because I feel that many things*, *we have to tell her*. *She is a girl child so*, *we have to tell her many things which she should be aware of and she should not be doing for her good…”* (Interview: M2, female parent, 38 years)

**Fig 3:**
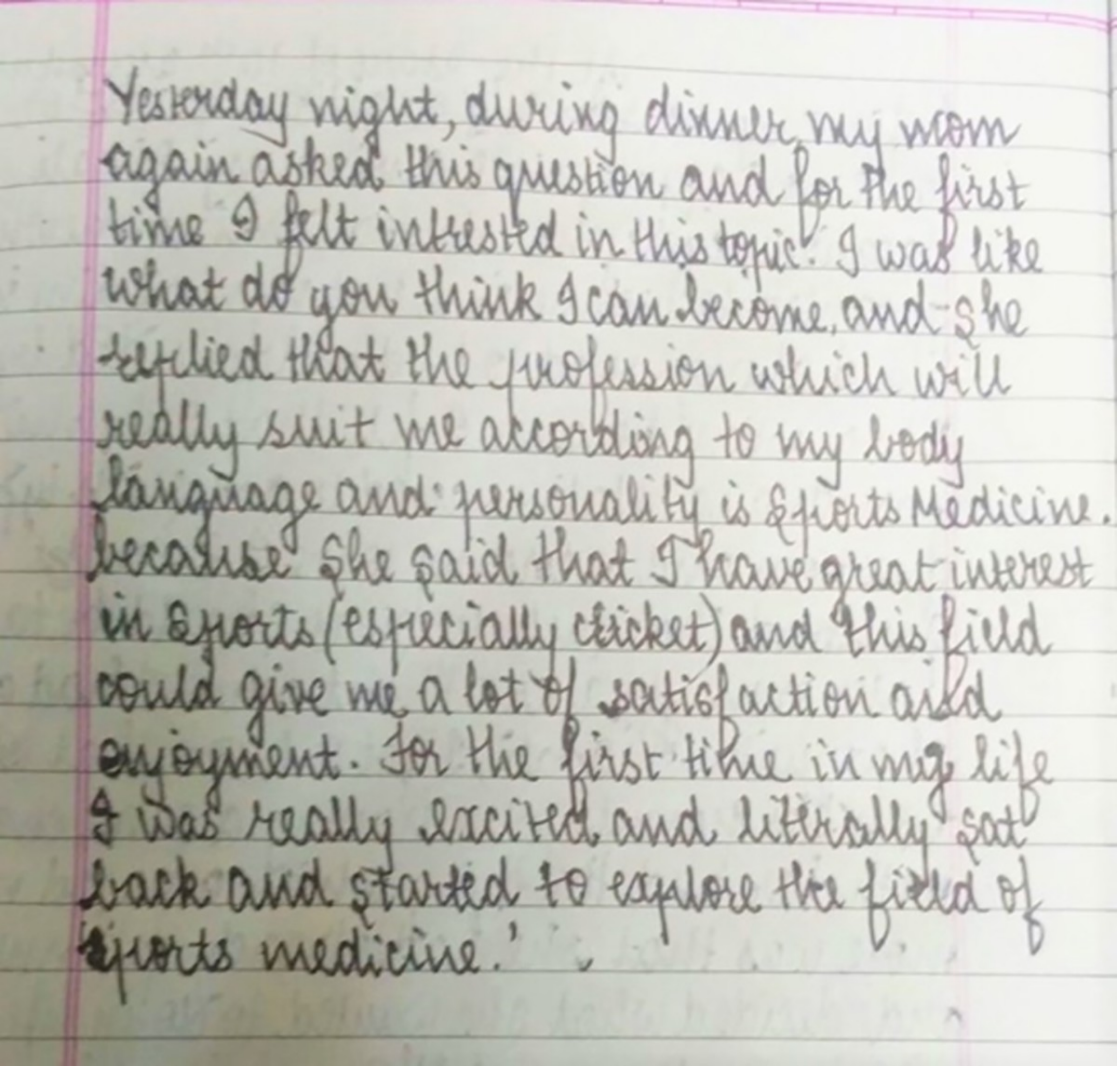
What do you think I can become?—Journal entry © G2, female, 15 years.

Despite these concerns, with regard to decision-making concerning career choices, this adolescent noted an incident in her journal regarding joint decision-making with her (Fig 3)

An excerpt from the journal entry of this participant states…*’I was really excited and literally sat back and started to explore the field of sports medicine*.*’* (Journal data: G2, female, 15 years)

Middle-adolescence is an important transitional phase where adolescents begin to make important career choices. This scenario of parents in involving the female adolescent making decisions about her career while keeping open routes of communication indicates a shift in the societal thought. We observed a difference in the provision and use of mobile phones between rural and urban female adolescents. Rural parents discouraged borrowing and using the parents’ phones. They held several concerns including the fear of their children engaging in online romantic relationships. One frustrated adolescent expressed her views:

*“My friends have their own phones*. *My mother owns a phone but she does not share the password with me as she feels it will distract me and waste my time but she gives the password to my younger sister*. *I just want to use WhatsApp and Facebook*, *that’s all… for use with friends*.*“*(Interview: G8, female adolescent, 16 years).

Traditionally held views of autonomy granting and family honour appears to have some continuity among families with female participants. To a large extent, however, our adolescent participants perceived that parenting behaviors including disciplining methods exercised by their parents stemmed from the concern they had towards the adolescent’s wellbeing. As one of our male adolescents observed:

“Our parents mold us. All the basic behaviors are all taught by parents and those things cannot be taught and cannot be influenced by friends. Parents, they teach you the right path and the wrong path. They are there for you.” (Interview: B3, male adolescent, 17 years).

These perceptions pave the way for better parent-adolescent communication and adolescent disclosure regarding their activities [56]. Albert I, et al., [57] in their study compared parenting between cultures and found that parenting including parental control meant ‘protection and care’ among Indian adolescents while it may be perceived as ‘constraint and overprotection’ in the western context. The inculcation of social norms was dependent on the cultural context and accepted as a normal part of development. Responsive and warm parenting was thus found to transmit from G1; however, subtle changes were observed in terms of an increase in granting age-appropriate autonomy to both male and female adolescents while maintaining lower levels of control in comparison with G1 which came through from our findings.

### Breaking the cycle of harsh parenting

The case study presented in Table 5 is a composite of our adolescent participants whose parents had experienced harsh parenting in G1. G2 participants largely lived in nuclear households and enjoyed closer relationships with their parents. G1 participants had made the decision to adopt warm methods of parenting.

**Table 5:** Case study– 2 perspective of an adolescent whose mother had experienced harsh parenting.

Amit (not his real name) is 16 years old and lives in an urban area in Karnataka, India. His nuclear household consists of his parents and an older brother. Both his parents worked in the city as teachers. He enjoys playing basketball and is actively engaged in school activities. In describing his relationship with his parents, he states that his parents had always allowed him to express himself since childhood. His parents would spend time with the boys and had been supportive of their sporting, academic activities and encouraged them to also pursue artistic endeavors. His mother would at times speak to them about the harsh environment in which she had been brought up but he had never seen his parents themselves engage in arguments or fights. Regarding disciplining, he stated that his parents would largely use verbal means. He had rarely experienced a beating when he was younger. These experiences made him feel secure and he held a lot of respect for both his parents.

“I tried not to interfere and I don’t think my wife has also been very strict. You know, from his (son’s) point of view, when we compare, he is having considerably more freedom.”

(Interview: F1, Male parent, 47 years)

Consequently, the use of warm parenting methods, encouraged his son to enjoy involved and positive parenting. He felt supported and shared family activities such as music.

**Fig 4:**
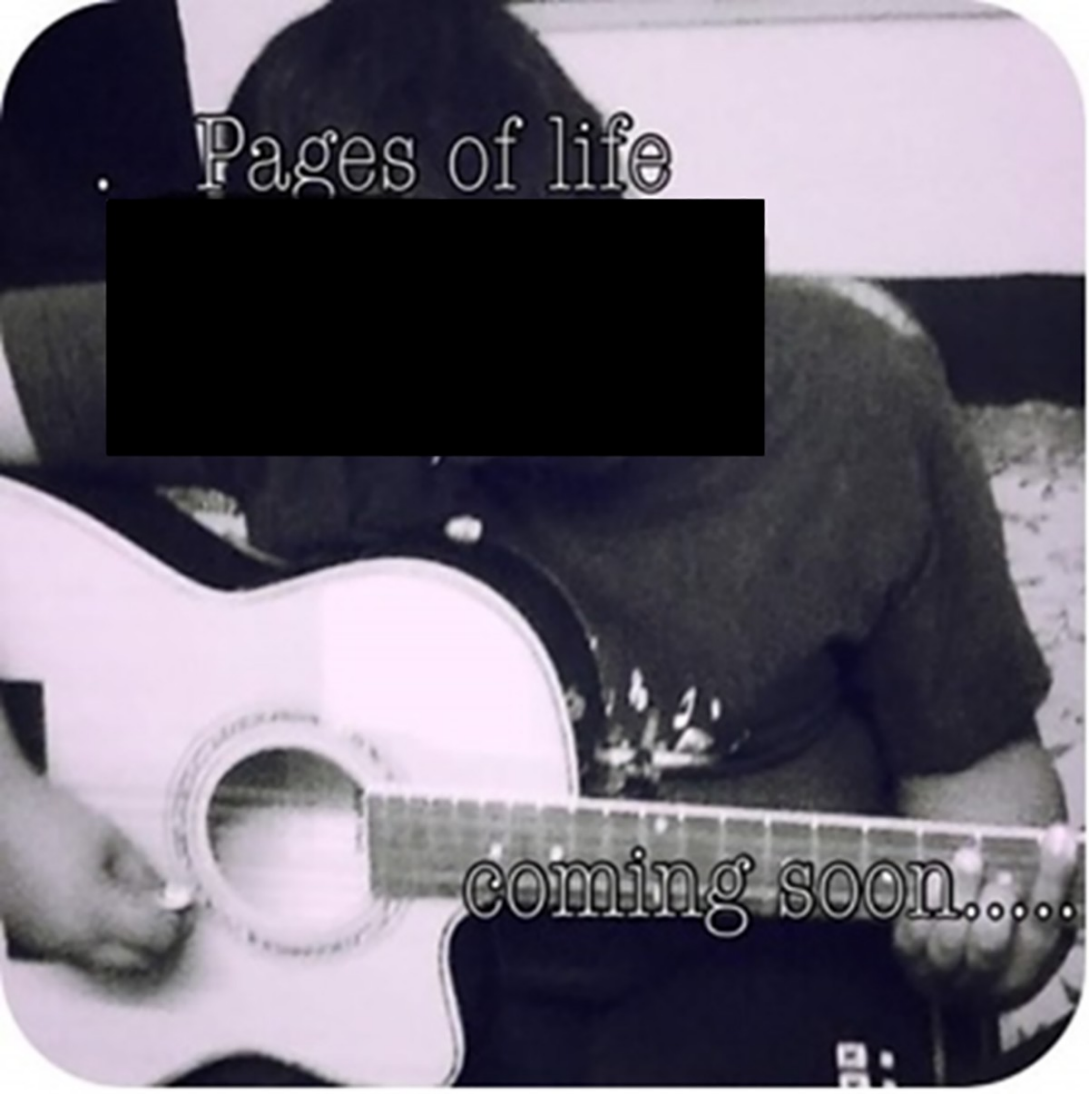
Follow your passion—Music © B1, male adolescent, 15 years.

**Fig 5:**
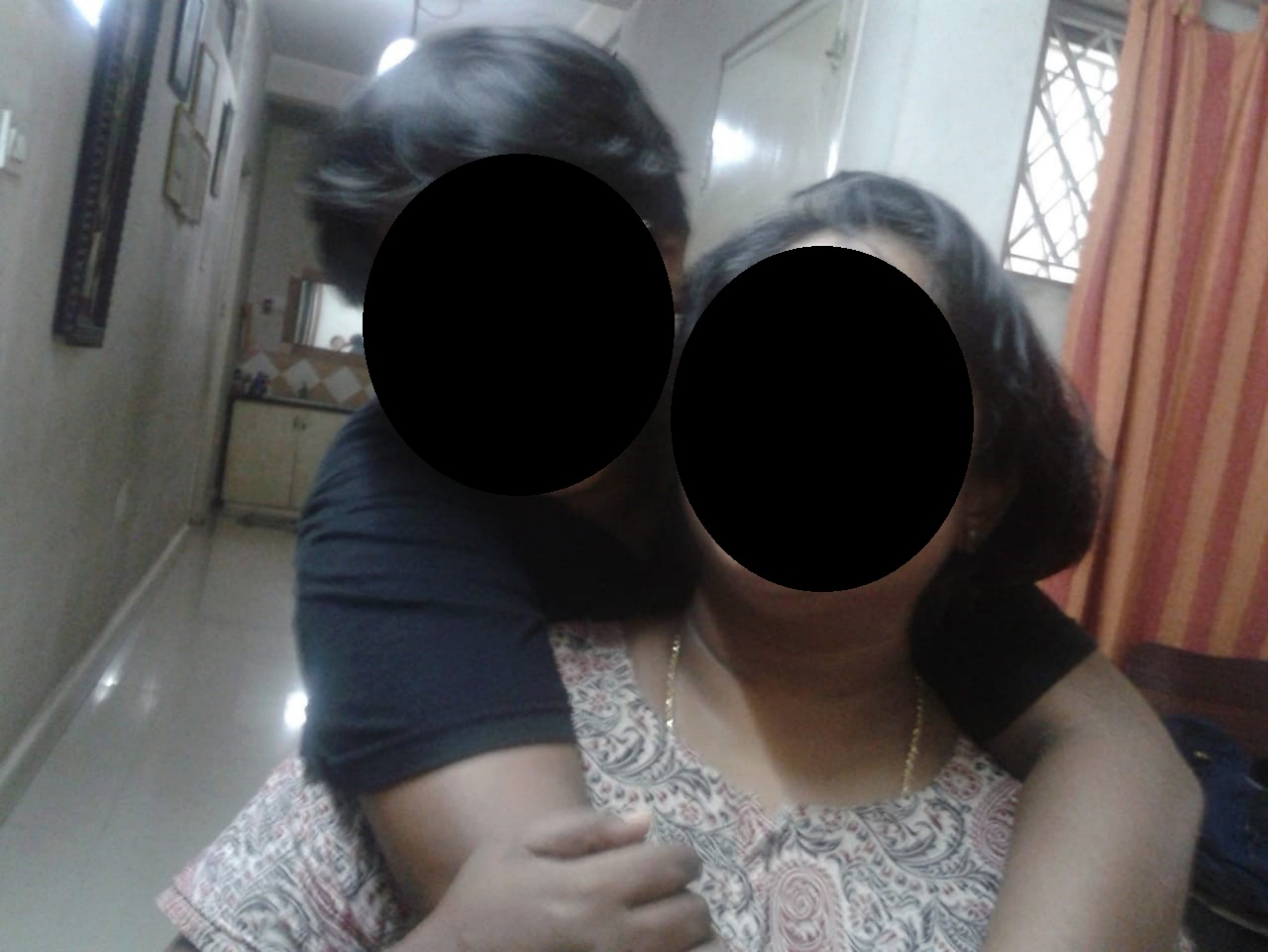
A moment with mom—© B1, male adolescent, 15 years.

*“I make my mom sit with me; she is my music critic*. *She gives me tips and points and she keeps asking me to make recordings*.*”* (Interview: B1, male adolescent, 15 years) Figs 4 and 5

Adolescents related that their parents preferred to discuss with them regarding matters of concern or used other verbal methods. As related by one of our adolescent participants:

*They use more verbal like… they just talk to me and shout at me or scold me…*.*they say*, *‘it’s not going to work out*, *that’s not right or… please don’t do it*.*’ Mom has tried the ‘silent treatment’ but it never worked*. (Interview: B1, male adolescent, 15 years)

**Fig 6:**
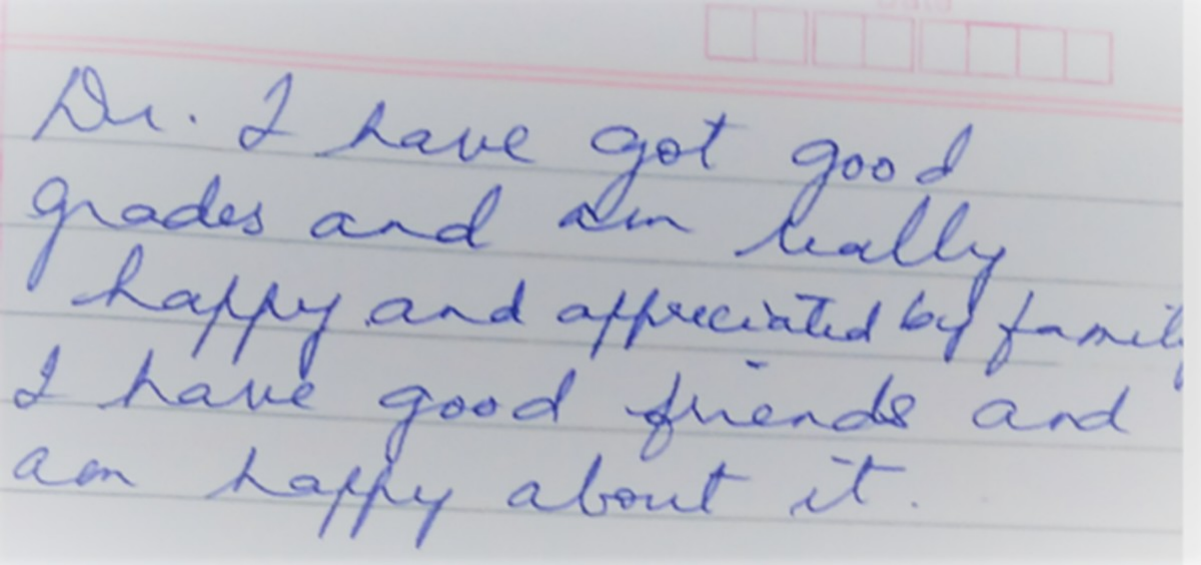
Appreciating family and friends—Journal entry © B9, adolescent male, 18 years.

We present the journal entry of the sons of a G1 parent who had experienced extremely harsh parenting. His entry states that he was ‘appreciated by family,’ achieved good grades and associated his life with happiness, indicating a psychologically adjusted adolescent. Our results find support in literature where the use of positive parenting methods are linked with optimal adolescent outcomes [51,56–60] (Fig 6).

Excerpt from journal entry: ‘*I have got good grades and am really happy and appreciated by family*. *I have good friends and am happy about it*.*’* (Journal data: B9, adolescent male, 18 years)

Data from his interview showed that he believed that his parents were ‘almost perfect’ because he could speak openly with them, they acknowledged his concerns and provide guidance.

*I think that they are almost perfect parents*. *I have the right to talk to them and I am sure*, *they will give me the right answer*. (Interview: B5, male adolescent, 18 years)

Establishing early and open communication routes between the parent and the child may be foundational in helping the adolescent have better outcomes. Thus, the findings of our study indicate that while warm parenting was transmitted across G1 to G2; the transmission of harsh parenting from G1 to G2 was interrupted with the intervention and support of both extended family and non-family community members in G1.

## Discussion

The family has been central to Indian society and has found mention in ancient texts [36]. Historically, family studies in India largely focused on patterns or types with little focus on dynamics and parent-child relationships. Post-colonial research has provided insights into family type, members that constituted the Indian household and types of families and have described the collective and interdependent relationships that define the Indian family [61].

Sociological research documents Indian families as being characteristically patriarchal with more than two generations living within the same household. Interdependence was emphasized as opposed to individualistic expression which is heavily influenced by collectivistic norms finding expression through family cohesion, belongingness and reciprocity founded on "collective responsibility" [36,62,63].

The post-colonial period has witnessed a change with industrialisation and urbanisation across the country and recent decades have witnessed these developmental changes occurring at a remarkable pace [63]. Observable changes have been occurring across several spheres in society and has percolated down to the family level. The National family health survey 2005 [64] showed an increase in the number of nuclear families concentrated in urban areas in comparison with national data previously. Other observable changes in the family structure, reduced age of the head of the household, an increase in female-headed households reflecting changes to traditionally held gender norms and sociocultural mores. However, these smaller units do continue to have strong bonds with their kith and kin and continue to extend support [37,42,65]. These changes could challenge and influence traditionally held views of family relationships and how individuals within the family interact with each other [66]. This study is an endeavour to capture the experiences of parenting across two generations. Specifically, we examined intergenerational transmission from the dual perspective of both parents and their adolescents.

Almost all of our parent participants in the G1 had lived in joint families but had moved to urban areas as nuclear units. G1 participants had experienced a range of different parenting experiences, from warm to extremely harsh parenting. The gender roles of their own fathers and mothers were according to the prevalent social norms in the Asian setting where fathers were providers and enforced discipline and were distant while mothers were the primary caregivers in all other aspects related to childrearing [64]. Extended families predominated in G1 and had included grandparents or other family members living within the same household. This structure may have led to the strengthening of familial bonds [36]; however, there may have been the dilution of individual parent-child dyad interactions. Warm parenting in the traditional context appeared to constitute meeting the needs of the family with a lower emphasis on communicating with or nurturing the interests of the adolescent. However, provisions were made for the adolescents in G1 to achieve adequate education from which to springboard into careers of their own. In terms of autonomy and disciplining, a distinct difference was observed between granting autonomy to male versus female adolescents. In contemporary society characterized by increasing urbanization and the breaking up of families into smaller nuclear units, the emphasis of strong bonds and kinship between the smaller units continue to be fostered [35,40]; however, parenting has changed in that parent-child interactions have increased as is evident from our findings.

G1 parents who reported warm parenting during their own childhood and adolescence preferred similar practices in parenting their children. The traditional Indian practise of restricting behaviours of female adolescents in keeping with gendered social disciplining norms was evident in G1 [37,68]. Contemporary parenting in G2 as gleaned from the multiple forms of data from our study indicates transmission of warm parenting. While the emphasis on education continues to remain unchanged, there appeared to be some differences in the nature of parenting with relation to the exercise of autonomy. The adolescent’s ability to exercise developmentally appropriate autonomy is always a function of parent-child relation. Adequate parenting helps the child to later have higher self-esteem as the taste of autonomy provides them with an opportunity to balance individuation with cooperation [40,58]. While both male and female adolescents were allowed to explore their interests and had the freedom to express their views, we were able to highlight changes related to autonomy for female adolescents between G1 and G2. Restriction of activities for female adolescent participants was less evident in G2 but was still enforced among some participants in rural households. Restrictions on female adolescents have been reported in rural areas and other urban settings in India in the past [67–70]. Hence, gender socialization continued to be reinforced in G2 in varying degrees. It was also interesting that in disciplining adolescents in G2, warm parenting in G1 did not obviate the belief that corporal punishment to a limited extent in G2 was acceptable. In keeping with the traditional cultural norms, however, the parenting methods adopted largely rested upon the father’s decisions [62,63]. The traditional hierarchical parent-child relationship appears to slowly be tempered with the observable changes in society including a definite rural to urban shift and rise in the urban middle-class while aspects of collectivism still continued to exist within this framework [63]. Overall, the description of what constitutes warm parenting appears to be evolving in contemporary India.

In the Indian context, corporal punishment has long-standing social sanction within Indian homes [71]. G1 participants experiencing harsh parenting stated that it was more often meted out by their fathers who abused substances, commonly alcohol use comparable with literature [72]. However, these parents, made conscious decisions not resort to the continuity of similar practices with their own children. Interruption in transmission of poor parenting behaviours between generations is possible in the presence of external support [27,34]. The memories of strong emotions such as fear, anger, shame and powerlessness that they had experienced as children surrounding the experience of harsh parenting were important driving factors among our participants. These memories coupled with access to social support from well-meaning non-family and extended family members who supported them during the harsh experiences, majorly influenced their decision not to adopt similar methods towards their own wards.

The emotional presence of parents is an important determinant of attachment and emotional regulation in later intimate relationships [27,73] and acts as a deterrent to poor adolescent outcomes [74,75]. There is a higher risk of developing long and short term sequelae ranging from physical to psychological disadvantages among those exposed to harsh parenting [27] without external support. The use of some psychological control methods to discipline adolescents also emerged which are documented in literature to lead to developmental trajectories that are not considered ideal [76]. However, this was not the primary form disciplining that was practiced. More often the adolescents were provided opportunities to talk with their parents through issues which may in the long term pave the way for better adolescent adjustment. The adoption of warm methods among our parent participants may hence, provide distinct developmental advantages to adolescents in G2.

The strength of our study has been in employing the multi-method approach which enabled us to draw rich perspectives from two generations and provided a glimpse into the lives of Indian families. We were able to delve into several subtle yet definite shifts in parenting across the two generations. Our findings brought to the fore preferred forms of parenting and transmission of parenting across generations, gendered differences in granting autonomy between male and female adolescents between generations, prevalent concerns of parents regarding female adolescents and more importantly, the laudable efforts of parents and the informal support systems that promote positive interactions among family members which prevented the vicious cycle of harsh parenting from transmitting to the next generation. Indian parenting has traditionally fit into the collectivistic parenting framework with value being placed on interdependence [42]. However, we were able to observe subtle elements of both collectivistic and individualistic values [62]—in the parenting behaviors towards G2. Exploring the parenting experiences and subsequent behaviors would add to literature on parenting dimensions and transmission of parenting in the Indian context perhaps lending to the development of indigenous parenting frameworks. To this end, our study has contributed to the theoretical deliberations on the changing face of the parent-adolescent dyad interaction in the Indian context.

With regard to the mixed use of qualitative and photovoice methodology, our study has been able to explore parenting through the parent-adolescent dyad perspective. The photovoice methodology has previously been found to be effective in research among adolescents [70,77]. This methodology has a strong participatory component, providing a deeper understanding of their inner perspectives through the use of imagery and narratives. The blending of photographs, journal and interview data and comparing it with qualitative data across two generations, provided access to unique perspectives which may have been otherwise difficult to achieve. Use of the photovoice method in family research may be underutilized. The broader application of this methodology as an assessment tool as well as a potential tool with therapeutic applications in the clinical setting needs further exploration among the younger population.

In conclusion, our study has attempted to probe into the continuity of parenting between generations. Our hope is to continue probing the various facets of Indian parenting and the influence it has on the development of adolescents within the indigenous cultural context.

## Limitations

This study did not explore parenting directly from the parents of G1 which may have added further understanding in achieving the study objectives. Given the qualitative nature of the study, to explore and gain insight into the parenting experiences of participants from two generations and the possible transmission across the two generations. The results are not intended to be generalized. There is a possibility of bias on the part of the participants including recall and social desirability response bias. The researchers had several measures in place to reduce social desirability bias. Firstly, the IDI guide was prepared with the guidance of social work, psychology and health professionals who assessed for the sensitivity of the guide.

Secondly, rigorous ethical requirements were met in maintaining confidentiality and anonymizing the data. Confidentiality aspects were discussed with the participants at the initial contact. For the photovoice component that the adolescents were participating in, they were given the choice of providing photographs of direct interactions with their parents or provide photos that indicated these interactions. The interviews using the photographs as prompts and the journal data supplemented this photo evidence. They were assured that there was no right or wrong answer and that we were open to listen to their experiences. Thirdly, the interviews were conducted by the first author, trained in conducting qualitative interviews and has herself has been a resource person in training researchers on qualitative methods. Lastly, the interviews were conducted in a place familiar to their participants, ie, their homes in a private room.

Future research on family relationships in the Indian context may benefit on focusing on other vertical and horizontal relationships including with grandparents and extended family members in the rural and urban settings were insufficiently explored in our study. Given the collectivistic bent of society in India, these relationships may provide further insight into how families function. Another important aspect that needs exploring are peer relationships which would lead to a richer understanding of peer relationships in shaping adolescent behaviours. These gaps in understanding need further probing to understand family relationships in our context.

## Conclusion

In conclusion, this study has brought to the fore unique cultural understandings that facilitate or deter the transmission of parenting between two generations specific to the Indian context. The UNICEF is co-custodian for 19 of the indicators under the SDGs [7] which may be achievable through policies which are family-focused as highlighted by the UNICEF [78]. The need for programs that equip parents with enhanced parenting skills and policies that promote positive parenting while reducing the use of harsh parenting cannot be emphasized enough. This may be key in promoting psychological adjustment among children and adolescents.

## Supporting information

S1 File Qualitative data excerpts—parents (G1). (PDF)

S2 File Qualitative data excerpts—adolescents (G2). (PDF)
