# Supplementary material for: ‘No, you should not beat our child because he will become aggressive:’ Applying a multi-method approach to explore intergenerational transmission of parenting practices
Source: PLoS One. 2021 Oct 7;16(10):e0258306. doi: 10.1371/journal.pone.0258306 (PMC8496842; doi:10.1371/journal.pone.0258306)
Supplement: S1 File — (PDF) [file pone.0258306.s001.pdf]

QUALITATIVE DATA EXCERPTS – PARENTS (G1)

**Interview excerpts**

**M7, female parent, 45 years**

**Excerpt 1**

I: About parenting, when you say warm what comes to your mind…what are the things you would say makes the atmosphere warm?

R: My mother used to …she was a warm person and she used to take care of us nicely ..do all the work and she was seeing each and everything. Father also used to, we had agriculture no, he used to see outside work and if we children need any particular things in school and all he looked into that…specifically going to like tour and all no, he used to send us all these things.

I: Would you be able to spend time with them on an every day basis?
R: Yeah yeah…in the evenings and all, we used to eat together, say prayers and all.

**Excerpt 2**

I: As you were growing up, if you felt you thought about things differently from your parents, would you be able to tell them, for example could you say 'I don't agree with this?' How would you go about that?

R: Yeah...sometimes I used to tell, but some things I was not able to tell them.

I: How about when you were older say about 15-16 years of age, how would your father respond if he did not like something you did?

R: He will shout and ……like clothes and if you wear some short short clothes and all, he will really shout: 'What you are wearing? Wear proper clothes'…like that.

**M3, female parent, 50 years**

**Excerpt 1**

I:  Could you please explain regarding your home atmosphere during your younger years.

R:  Everything was there enough...so for us eating was not a problem.  It is available about thinking about us was minimum because, it was not necessary at that time to think for children. The more focus was on agriculture, production, those things and slowly all these my grandchildren became older so my mother's responsibility was to take care of all these four people, their deaths, everything, their hospital, she was busy with these things.

I:  And how old were you when all these things were happening?

R: This was less than 13-14 years till about 15 years.  I think all three grandparents died and the fourth one but of course after 8th.  I was shifted to hostel I grew up in the hostel, 8th, 9th, 10th, PUC…I was in my uncle's house…so there was no importance for us at all…even my desire, my interests were never taken care of and in the hostel again…I did'nt like the life there…it was very isolating, very boring it was…even childhood was not very good.  Father used to love me so much but he had no time to give me.

**F4, male parent, 51 years**

**Excerpt 1**

I: And towards you like if you did something good in school, would you be appreciated by your father?

R: Since we were five children, there was not much acceptance…you know…whenever we did something well…when it came …he will say good, that's all.

**M2, female parent, 38 years**

**Excerpt 1**

I: Could you describe how your parents were towards you during your growing years?

R: My mother was strict actually. My father was also strict. Strict in the way that they allowed me to do whatever I wanted to do but they would tell me what to do. It was a normal household with elders and all. We were not many children, me and my brother were there. We were not allowed to go anywhere and all….that was the thing. Otherwise, not very strict. Father was very strict, he was very strict with our studies. That I didn't like. That is the only thing. Otherwise, he was a nice person actually.

**Excerpt 2**

I: Do you feel that sometimes, if you disciplined her and did not allow her to do certain things does it lead to conflict or does she agree to it…how does she respond?

R: No, until she was in the 8$^{th}$ standard ….that time it was there actually ….now she understands, she is grown up no...since last few months. I am finding she has changed actually. I tell her also, she understands. I may be correcting her but she understands and she will say…ok …she is not much worried about that now. Otherwise, she used to get angry (laughs). If I tell her to do anything, she will..what to say be arrogant, lot of anger, now it is not like that. She has changed now, may be they also understand as they are growing up.

**Excerpt 3:**

I: Do you feel you and your husband agree mostly on how to discipline your daughter?

R: Sometimes, I will have to disagree with him ….because I feel that many things….we have to tell her. She is a girl child no….so, we have to tell her many things ….she should be aware of and she should not be doing for her good only.

**F5, male parent, 55 years**

**Excerpt 1**

I: How would you describe your father's relationship with you? Was he encouraging or strict towards you?

R: He used to talk to me. There was no problem….. I ….I had more liberty compared to my brothers and sisters …as I was the eldest. He used to appreciate me more.

**Excerpt 2**

I: How do you think about how you parent your daughter? Do you feel you allow your daughter to express herself or do you feel you need to tell her how to go about things?

R: Ya, am like that…I am like that. Even when I was growing up it was like that, my father was like that. So I just try to do the same thing with her… I want to bring up my daughter in the same way. I don't force her for anything…like…whatever she wants like…I am sort of friendly with her.. I just wanted her to be like a friend. That's why, I encourage her in everything. First of all, I wanted her to become good because I am not much worried about her academics. She speaks to me also, all things..what's happening in school, everything she tells me. I won't force her …even for her studies or anything like that. I am not expecting discipline and all. She is okay. She is very confident. When I see that, I feel very happy, like what I couldn't do, she is doing that.

**Excerpt 3**

I: How about disciplining? Sometimes you may have to teach her certain things…how do you go about it?

R: Yean…because sometimes it happens…like in this age, sometimes she may be a bit demanding then, in a friendly way, you make her understand, not a lot of shouting or anything like that.

**M1, female parent, 44 years**

**Excerpt 1**

I: As you were growing would you be able to make decisions on your own…for example regarding your clothing, etc.?

R: I liked wearing jeans you know which he (father) doesn't like. He does not tell me now…but I very, very clearly remember, he did not like me wearing jeans. So, I had not worn it for a long time. Once, I got married, then there were no issues."

**Excerpt 2**

I: Regarding disciplining your son, how do you generally go about it?

R: I don't know….. I am not as patient as I do get angry and I do use some strong words once in a while. I never physically, what you call beat my son…. may be once or twice. Simply because my husband and mother-in-law did not believe in hurting a child. You know, we have all been beaten. If I was not studying, my mother used to beat me with a scale, belt also, because she wanted to make me understand ……so ….but she has never gone overboard to the extent that I became aggressive. But my husband told me: no, you should not beat our child because he will become aggressive….he says when his father had beaten him, he felt very aggressive and wanted to beat him back. I believe that, once in a while, you need to teach him certain things...

**M9 female parent, 52 years**

**Excerpt 1**

I: Could you describe your home atmosphere when you were younger?

R: Our home atmosphere…there was no good relationship between my father and mother which definitely had an impact on me…..also, on my siblings but me …..basically because …..maybe I was an older child ….I always reacted. My father's unmarried older sister had a real influence in making decisions…it could be regarding our academics and whether we should get involved in extra-curricular activities. So, very much restricted timings to get back and we were not allowed to …..socialize. He would drink, he would abuse us verbally, he would even hit my mother …and the neighbors would come to help us and we would feel so ashamed. Sometimes, we would hide our mother. Generally also, I have seen certain things which are not good to see between my parents. The way sometimes …..he had looked at me was not in the right way….I would escape. And.. nobody knows about it, even my mother…. I could not speak about it to her. I was so angry and wished he was dead. These were all issues that really troubled me.

**F2, male parent, 46 years**

**Excerpt 1**

I: So, about your parents, your father was he encouraging or was he strict with you?

R: No, no, he was never strict with me. He was extremely friendly person though the age gap between me and my father was huge…like…even my parents' age gap was around 18-20 years. Ya, but actually main thing was…my father's influence was very good like..even though I lost my father, when I was in 10$^{th}$ standard but until then, he was there to guide me and like the thing was…right way…that was for me a big influence. My father was almost 50. It was a late marriage so, in spite of that, it didn't come in the way…like this…he was a very friendly person, never used to scold me or doing anything like that.

**M4, female parent, 46 years**

**Excerpt 1**

I: Do you feel your parenting is similar or different from the way you were brought up as a child?

R: He is always having the freedom…I have always given him the freedom to come and talk to me about whatever happens whether it is positive, negative anything, whether it is right, wrong … I am always telling that it is okay but you have to come and disclose it to us as parents, we should know that it is rather than some outsiders coming and telling me, I should always know it from you. This is what I have been telling him that we should know. He is scared that I would angry…that way….but later on he tells me…then I ask him, why you did'nt tell me earlier? But most of the time, he tells me everything that way. We have to be very close to our children. We have to understand them because this is the age when they need somebody close and in his case, he is an only child…. we try to give him the support in whatever way we can.

**F1, Male parent, 47 years**

**Excerpt 1**

I: In your younger years, what was the kind of discipline used by your father towards you?

R: My father was strict, my father believed in corporal punishment.. if I made a mistake. He would use his belt ….when I was bullying my sister or something…first belt, then talk. Actually, right up to my teenage…..and that was enough. I was punished as a child, so that was the reason why when it came to our parenting, there are some ground rules. Even when my wife violates them, sometimes I feel angry. I am completely against any sort of physical punishment. I have never beaten my child, never….. except probably once. So, I would probably say that was the only ground rule which I think, I used for parenting. I tried not to interfere too much ….and I don't think my wife has also been very strict. You know, from his ..my son's point of view, when we compare, he is having considerably more freedom.
