# Supplementary material for: ‘No, you should not beat our child because he will become aggressive:’ Applying a multi-method approach to explore intergenerational transmission of parenting practices
Source: PLoS One. 2021 Oct 7;16(10):e0258306. doi: 10.1371/journal.pone.0258306 (PMC8496842; doi:10.1371/journal.pone.0258306)
Supplement: S2 File — (PDF) [file pone.0258306.s002.pdf]

QUALITATIVE DATA EXCERPTS – ADOLESCENTS (G2)

Interview excerpts

B1, male adolescent, 15 years

Excerpt 1

I: Could you please explain what this picture is about ….where you are with your guitar?

R: This picture was originally intended and is kind of the original album which I started to work on and I can't say that it has only original scores cause I haven't even started writing one but what it is ……..is it is the cover photo of my album my music album. The one which I intend to fill up on. only one song that I have uploaded so that picture number 1. This was taken just on the spot. I thought why not I take it now. And I had the iPad and the photo quality was so bad I thought if I put it in black and white it will look a bit 70 or 80 ish so that people think it's not bad quality of photo he has edited on purpose so that's picture number 1.

I: Can you tell me something about what you think about music and how important it is for you?

R: Well like I told you last time music is something which I when I am really upset or I don't feel like talking to anyone or I just want to rest most of the time. It's on my phone when I ….when I rest like ….I just keep meddling with my phone. What I'd like to do is I just like to fidget around with something and play music on my speaker and listen do it for as long as I can and if I am really bored I just take the guitar and play along and learn a new song and if there are competitions and all then I tend to rigorously work on it.

I: What kind of competitions?

R: Music talent shows I have gone. If you considered intra school I have done only 2 which was in Chennai when I was living there yeah ….unfortunately, in school I wasn't allowed to use instruments and I sang and it was only Indian or classical because of which I wasn't able to do what I could normally no instruments allowed.

R: Then there was this competition called Campus Idol so that I could sing and play an instrument and I was allowed to use karaoke so in those kind of situations I tend to listen to a lot more music and sing along but of course I take it pretty lightly ….it's a passion more of a you know a force.

I: And, your parents are okay with this?
R: Yeah my mum in fact she is so enthusiastic about me and my music she is like she wants me to go to an institute of music and work even more on it and all that kind of stuff.

I: What does your father have to say?
R: Dad like he thinks I can but he doesn't believe like you know ….so his thought is not everyone is a (famous singer’s name) which is pretty true you can't become very famous like him. If you want to do well in music, then you have to practice and go for classes and all that and without classes, I mean without practicing ….it’s not this goal. And whether or not you become famous… if it is your passion you will have to keep working on it… so that's what is his opinion and similarly its is what mum is also saying. They are pretty supportive about my music.

I: Do you have restrictions that you have to do within a certain time or something like that?

R: Nothing like that in fact more than me ….like whenever I take a break I tend to be on my phone, so mum ….my mum prefers, like both mum and dad…they prefer that I am listening to music rather than this thing. Everyday morning, especially when my dad is there, mum plays the music channel on TV and we just keep doing our work or I'll connect my phone to the speaker and I listen to music so that you don't realize time moving so that's my connection with music. So… I am encouraged to pursue music.

Excerpt 2

I: How do your parents generally discipline you?

R: They use more verbal like they just talk to me and shout at me or scold me or emotionally blackmail me. They go looking like really sad and not talk to me or saying, 'it's not going to work out, that's not right' or 'we are okay the way you are so, this is what you give us in return! Please don't do it.' Mom has tried the 'silent treatment' but it never worked.

I: Have you heard of corporal punishment being used on adolescents?

R: So, very recently when I was doing my 10th grade around my 4th formative assessment test, there was a friend of mine. He lives here only in the neighbourhood. What happened was he came and he got relatively low marks. And, he is very silent and he is more on the introvert side. He doesn't talk a lot and no one talks to him a lot. He just sits in a corner and does his own work. No one knows what he does also and I asked what his marks were. He got pretty low marks. So for 90, he got 28 or something. He said, 'My mom hits me with my belt' and all that. According to me, that's not very good. It sounds a bit philosophical because what I am saying is for us at our age, more than physically scarring us, it emotionally scars us that someone whom we thought would support us, is coming and hitting us. I don't even know how he reacts because his face is so neutral when he talks. He is so emotionally dead from the inside that he just doesn't react.

G2, female, 15 years

Excerpt 1

Interviewer: So tell me about your dancing..

Interviewee: It’s my passion… like I love it.

Interviewer: Alright so what form of dancing is this? when did you start?

Interviewee: I started this dance form ‘Mohiniyatam’ when I was in senior kindergarten.

Interviewer: Ok and you have given many performances, yes?

Interviewee: Yeah

Interviewer: Alright…. have you thought of it as a profession or it’s just a hobby?

Interviewee: No… it’s just making me feel happy and encouraged…. like any dance form.

Interviewer: Alright ..so you do you always feel that you have always been encouraged to do this?

Interviewee: Yeah, my parents encourage me to dance.

Interviewer: Ok how about in this year you know in your 10th grade…were you able to do concentrate on dancing you feel?

Interviewee: It was not like no one encouraged me ….all said that you can go but then, my dance classes and tuition classes timings clashed. So, I had to stop it.

Interviewer: Ok what did you feel at that time?

Interviewee: Like my whole year was like ….I didn’t like 2017 to be frank because it was only school, tuition, homework and all were like its time just to study after study.

Interviewer: So, this year was very hectic right? So, do your friends dance as well or its just you?

Interviewee: No …my friends also dance

Interviewer: The two of them you are close with ?

Interviewee: Yeah ... correct

Interviewer: Ok the same dance?

Interviewee: No, no …they do Bollywood

Interviewer: Ok is that one of the reasons you are close to them, that you all like dancing?

Interviewee: No may… .maybe a…it is because I feel comfortable with them like. Earlier also when we were not close, if I were low they used to come to me.

Journal data – G2, female, 15 years

DATE:

Yesterday night, during dinner, my mom again asked this question and for the first time I felt intrested in this topic. I was like what do you think I can become, and she replied that the profession which will really suit me according to my body language and personality is Sports Medicine. because she said that I have great interest in sports (especially cricket) and this field could give me a lot of satisfaction and enjoyment. For the first time in my life I was really excited and literally sat back and started to explore the field of 'sports medicine.'

G8, female adolescent, 16 years

Excerpt 1

I: Do you own a phone?

R: No

I: Do you wish to own a phone?

R: Yes

I: Do your friends own phones?

R: Yes, everyone has phones. The other day they brought it for talents day. I was the only one without it.

I: Is there any restriction in your college against phone use?

R: On certain days you can bring it. When they say you can take it with you, they let you know.

I: Do they own these phones or are these their parents' phones?

R: It's their own phones. my mother owns a phone. she does not share the password with me as she feels it will distract me and waste my time. But she gives the password to my sister.

I: Why do you wish for phone for yourself?

R: Just for WhatsApp and Facebook that's all. For use with friends.

B3, male adolescent, 17 years

Excerpt 1

I: Do you think adolescents need to be disciplined by parents?

R: That's like, I think they are trying to make me a better person. I know it, I know what they do is for my betterment. It's for my own good. It's good. What I think is that there must be some discipline because adolescents are, you know at their age, they have like so many friends. If they don't get love or the care they want, the discipline they want, if they don't get that in time or like, sufficient enough, by that time they deviate to rude behaviour, like smoking and drinking like that and all. So, I think there should be awareness among the adolescents.

Excerpt 2

I: And what do your parents feel about your friends you think?

R: When you are out with your friends and you know, when you have someone you can rely or count on them, if you have good company, like a lot of best friends, it's okay but then in bad company, you get influenced or you have influence. Then, it also comes down to how parents teach, guide the children, moulding them in teenage. So, a student's or a child's behaviour does not depend only on their parents but it depends on their friends also.

I: Would you rely more on your parents or friends to help you know what choices to make?

R: Our parents mold us. All the basic behaviors are all taught by parents and those things cannot be taught and cannot be influenced by friends. Parents, they teach you the right path and the wrong path. They are there for you.

Journal data - B9, adolescent male, 18 years

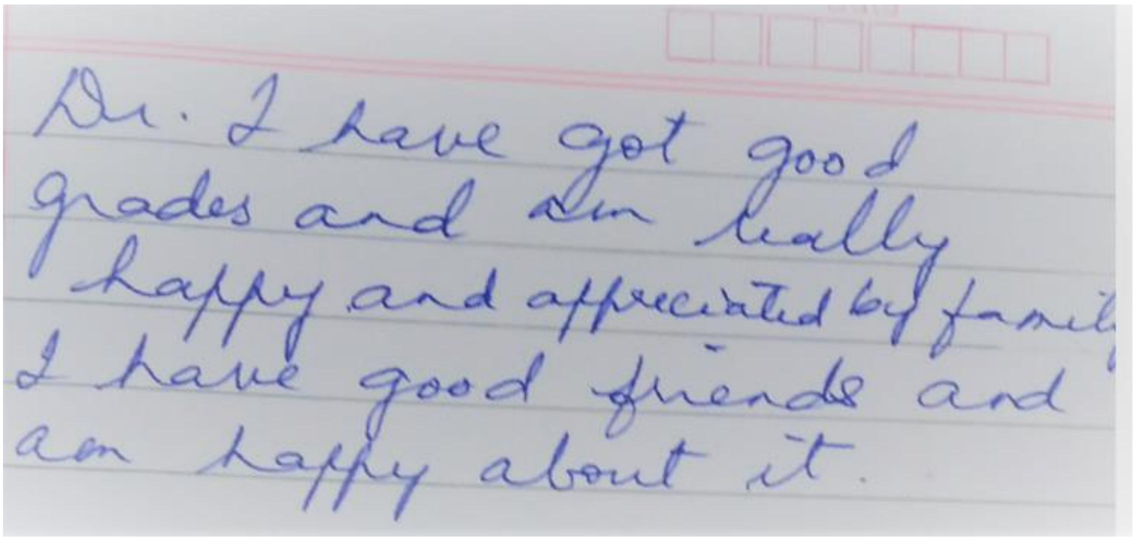
Dr. I have got good
grades and Am really
happy and appreciated by famil
I have good friends and
am happy about it.

B5, male adolescent, 18 years

Excerpt 1

I: How would you describe your relationship with your parents overall?

R: I think that….. they are almost perfect parents, not much problem with them. I usually don't have a problem with what I want. I don't ask for many things but what I like, they try giving me, be it anything, be it some sports equipment or food or if I want to go out with friends. They usually don't object. I feel it's a very good. And, even like if I have problems, may be any kind of problem, in school or something, I have the right to talk to them and I am sure, they will give me a right answer. So, I can discuss with them.
